# Prevalence and risk factors of fatty liver disease in psoriasis: A systematic review and meta-analysis based on NAFLD/MAFLD diagnostic criteria

**DOI:** 10.1016/j.jdin.2026.03.009

**Published:** 2026-03-26

**Authors:** Jiayu Shen, Liu Liu, Jiao Wang, Xiaoce Cai, Miao Zhang, Qingyun Wang, Xin Li, Xiaoying Sun

**Affiliations:** a Department of Dermatology, Yueyang Hospital of Integrated Traditional Chinese and Western Medicine, Shanghai University of Traditional Chinese Medicine, Shanghai, China; b Institute of Dermatology, Shanghai Academy of Traditional Chinese Medicine, Shanghai, China

**Keywords:** MAFLD, meta-analysis, NAFLD, prevalence, psoriasis

*To the Editor:* Previous studies have established a close association between psoriasis and nonalcoholic fatty liver disease (NAFLD).^1^^,^^2^ Psoriasis and NAFLD share key pathophysiological features, including systemic inflammation and insulin resistance, which can contribute to a mutually exacerbating cycle.^3^^,^^4^ In recent years, the introduction of new inclusive diagnostic criteria has updated the definition of metabolic dysfunction-associated fatty liver disease (MAFLD), which has gradually replaced NAFLD in clinical practice across most countries and regions.^5^ No study has yet explored the link between fatty liver disease and psoriasis using both NAFLD and MAFLD criteria or examined key modifiers in depth. Our meta-analysis fills this gap by determining the prevalence of NAFLD/MAFLD in patients with psoriasis and evaluating differences across key subgroups, thereby identifying factors associated with higher co-prevalence and provide updated evidence.

We searched the PubMed, Embase, Cochrane Library, and Web of Science databases from inception through July 2025, eligible trials included observational studies reporting NAFLD/MAFLD prevalence in psoriasis patients. Psoriasis diagnosis had to be standardized, and NAFLD/MAFLD diagnosis required clear criteria. Controls consisted of patients without psoriasis. A random-effects model determined pooled prevalence, the odds ratio and 95% confidence intervals (CI) with R software (version 4.4.2; R Foundation for Statistical Computing). Meta-regression based on study-level aggregate data were performed to assess the potential influence of characteristics on the estimated prevalence. Subgroup analyses were conducted for variables that were statistically significant in meta-regression or considered clinically important a priori, ensuring clearer and more robust interpretation of the findings. Findings derived from this standard meta-analysis are reported as pooled estimates. Results from meta-regression are reported as regression coefficients (β value) with 95% CI and *P*-values. Detailed information on the methodology is provided in the Supplementary Data, available via Mendeley at https://data.mendeley.com/datasets/9rswkdvyjk/2.

Our meta-analysis of 52 studies (Supplementary Fig 1 and Table I, available via Mendeley at https://data.mendeley.com/datasets/9rswkdvyjk/2) involving 349,490 psoriasis patients found a 38% (95% CI 0.32-0.44) pooled prevalence of NAFLD/MAFLD. Geographic variation in the comorbidity prevalence was presented in Fig 1. Higher prevalence was observed in males (51%, 95% CI: 0.45-0.57), cross-sectional studies (43%, 95% CI: 0.36-0.49), studies with sample sizes of 100-500 (45%, 95% CI: 0.38-0.52).The highest prevalence was identified using transient elastography (63%, 95% CI: 0.43-0.83). Using MAFLD or NAFLD as the diagnostic criteria yielded similar prevalence (40% and 38%, respectively) (Table I, Supplementary Figs 2 to 9, available via Mendeley at https://data.mendeley.com/datasets/9rswkdvyjk/2). These subgroup findings were consistent with the meta-regression results (Supplementary Table II, available via Mendeley at https://data.mendeley.com/datasets/9rswkdvyjk/2).

**Fig 1:**
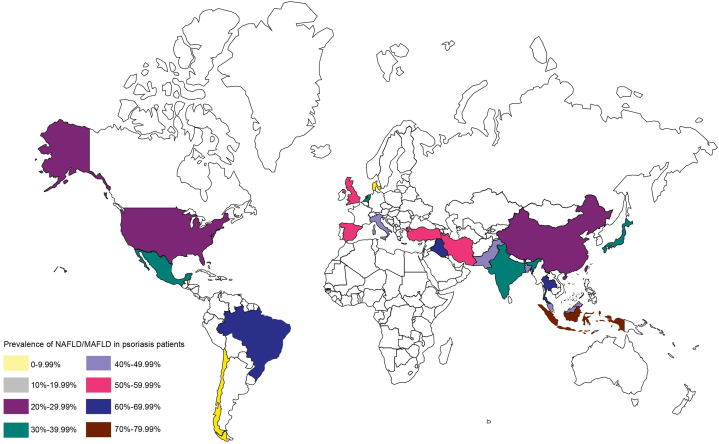
Worldwide prevalence of NAFLD/MAFLD among patients with psoriasis. Number (prevalence) of studies included per region: Asia, *n* = 28 (37%); Europe, *n* = 15 (45%); North America, *n* = 7 (29%); South America, *n* = 2 (27%). *MAFLD*, Metabolic dysfunction–associated fatty liver disease; *NAFLD*, nonalcoholic fatty liver disease.

**Table I:** Pooled prevalence of comorbid NAFLD/MAFLD in psoriasis patients

Study or subgroup	Prevalence [95% CI]	I^2^	*P* value
Overall prevalence	0.38 [0.32-0.44]	99.4%	<.001
Sex			
Male	0.51 [0.45-0.57]	81.7%	<.001
Female	0.37 [0.32-0.43]	68.4%	<.001
Region			
Europe	0.45 [0.36-0.54]	99.3%	<.001
Asia	0.37 [0.29-0.46]	99.5%	<.001
North America	0.29 [0.15-0.44]	98.5%	<.001
South America	0.27 [0.00-0.61]	97.5%	<.001
Study design			
Cross−sectional study	0.43 [0.36-0.49]	99.4%	<.001
Case−control study	0.35 [0.24-0.46]	98.7%	<.001
Cohort study	0.11 [0.00-0.28]	99.7%	<.001
Psoriasis severity			
mild	0.13 [0.00-0.37]	94.7%	<.001
moderate-to-severe	0.29 [0.28-0.51]	99.5%	<.001
Sample size			
<100	0.40 [0.32-0.49]	93.8%	<.001
100∼500	0.45 [0.38-0.53]	97.7%	<.001
>500	0.13 [0.01-0.25]	99.7%	<.001
Diagnostic methods of MAFLD			
Ultrasonography	0.43 [0.37-0.48]	96.9%	<.001
Transient elastography	0.63 [0.43-0.82]	94.8%	<.001
CT	0.31 [0.19-0.43]	66.7%	.08
USFLI	0.35 [0.27-0.43]	/	/
MRI	0.48 [0.34-0.63]	/	/
Guidelines	0.31 [0.04-0.58]	99.5%	<.001
Past medical history	0.14 [0.03-0.24]	99.7%	<.001
Diagnostic Criteria of fatty liver disease			
NAFLD	0.38 [0.32-0.44]	99.4%	<.001
MAFLD/MASLD	0.44 [0.31-0.57]	99.6%	<.001

*CI*, Confidence interval; *CT*, computed tomography; *MAFLD*, metabolic associated fatty liver disease; *MASLD*, metabolic dysfunction-associated steatotic liver disease; *MRI*, magnetic resonance imaging; *NAFLD*, nonalcoholic fatty liver disease; *USFLI*, US Fatty Liver Index.

Compared to the nonpsoriasis control group, psoriasis patients had a significantly higher odds of NAFLD/MAFLD (OR: 2.23, 95% CI: 1.86-2.68, Supplementary Fig 10, available via Mendeley at https://data.mendeley.com/datasets/9rswkdvyjk/2). Furthermore, psoriasis patients with comorbid fatty liver disease also exhibited higher Psoriasis Area and Severity Index scores (weighted mean difference: 4.29, 95% CI: 2.54-6.05, Supplementary Fig 11, available via Mendeley at https://data.mendeley.com/datasets/9rswkdvyjk/2).

Our extensive analysis maps the psoriasis-NAFLD/MAFLD comorbidity landscape and highlights that demographic, geographic, and diagnostic factors influence prevalence, with a strong pathophysiological link evident in more severe symptoms among comorbid patients. There are some limitations. Variability in diagnostic criteria for NAFLD/MAFLD and psoriasis severity across studies introduces population differences. Reliance on medical history for the diagnosis of NAFLD may risk underdiagnosis, potentially leading to an underestimation of the true association. Publication bias and the predominance of data from high-income regions limit the global relevance of the findings. Our findings highlight the need for regular NAFLD/MAFLD screenings and effective metabolic management in psoriasis patients, advocating for multidisciplinary efforts to improve outcomes.

## Conflicts of interest

None disclosed.
